# GTSE1, CDC20, PCNA, and MCM6 Synergistically Affect Regulations in Cell Cycle and Indicate Poor Prognosis in Liver Cancer

**DOI:** 10.1155/2019/1038069

**Published:** 2019-12-30

**Authors:** Yongchang Zheng, Yue Shi, Si Yu, Yuanyuan Han, Kai Kang, Haifeng Xu, Huajian Gu, Xinting Sang, Yang Chen, Jingyu Wang

**Affiliations:** ^1^Department of Liver Surgery, Peking Union Medical College Hospital, Chinese Academy of Medical Sciences & Peking Union Medical College, Beijing 100730, China; ^2^Center of Tree Shrew Germplasm Resources, Institute of Medical Biology, The Chinese Academy of Medical Sciences and Peking Union Medical College, Yunnan Key Laboratory of Vaccine Research and Development on Severe Infectious Diseases, Kunming, Yunnan 650118, China; ^3^Department of Hepatobiliary Surgery, Affiliated Hospital of Guizhou Medical University, No. 28, Guiyi Street, Yunyan District, 550004 Guiyang, China; ^4^MOE Key Laboratory of Bioinformatics, Center for Synthetic and Systems Biology, Bioinformatics Division, BNRist, Department of Automation, Tsinghua University, Beijing 100084, China; ^5^Department of Basic Medical Science, Tsinghua-Peking Joint Center for Life Sciences, School of Medicine, Tsinghua University, Beijing 100084, China

## Abstract

GTSE1 is well correlated with tumor progression; however, little is known regarding its role in liver cancer prognosis. By analyzing the hepatocellular carcinoma (HCC) datasets in GEO and TCGA databases, we showed that high expression of GTSE1 was correlated with advanced pathologic stage and poor prognosis of HCC patients. To investigate underlying molecular mechanism, we generated GTSE1 knockdown HCC cell line and explored the effects of GTSE1 deficiency in cell growth. Between GTSE1 knockdown and wild-type HCC cells, we identified 979 differentially expressed genes (520 downregulated and 459 upregulated genes) in the analysis of microarray-based gene expression profiling. Functional enrichment analysis of DEGs suggested that S phase was dysregulated without GTSE1 expression, which was further verified from flow cytometry analysis. Moreover, three other DEGs: CDC20, PCNA, and MCM6, were also found contributing to GTSE1-related cell cycle arrest and to be associated with poor overall survival of HCC patients. In conclusion, GTSE1, together with CDC20, PCNA, and MCM6, may synergistically promote adverse prognosis in HCC by activating cell cycle. Genes like GTSE1, CDC20, PCNA, and MCM6 may be promising prognostic molecular biomarkers in liver cancer.

## 1. Introduction

Liver cancer is one of the most common causes of cancer mortality around the world. Hepatocarcinogenesis is frequently associated with gene dysregulation, which may be influenced by risk factors including hepatitis virus infections, toxic exposures, alcohol consumption, and obesity [1]. Due to progress in diagnosis and surgical techniques, recent improvements have been observed in patients' outcome. However, lack of effective prognostic indicators greatly limits the overall survival. Therefore, identification of prognostic markers is critical for improving liver cancer treatment.

*GTSE1* is a p53-inducible gene mapped to chromosome 22q13.2-q13.3 and is expressed explicitly during the G2 and S phase of cell cycle [2, 3]. GTSE1 proteins mainly localize on microtubules and regulate microtubule dynamics by inhibiting the microtubule depolymerase MCAK, which is critical for chromosome stability, accurate alignment, and segregation [4]. Besides, it also regulates cytoplasmic localization of p53 with its characterized nucleocytoplasmic shuttling ability [5]. After DNA damage, GTSE1 is required for G2 checkpoint recovery by regulating p53 localization, stability, and function [6, 7]. While in normal condition, *GTSE1* is activated by functional p53 binding on the *GTSE1* promoter region and encodes GTSE1 protein to cause a delay from G2 to M transition [3, 8]. Recent studies revealed that overexpression of GTSE1 contributes to promoting cell proliferation, migration, and invasion in different cancers via translocalizing p53 to the cytoplasm [9, 10].

High expression of GTSE1 was associated with short overall survival of patients suffering breast cancer, bladder cancer, and/or liver cancer [9–13]. Also, elevated GTSE1 significantly interferes with chemotherapy efficacy and influences the survival probability of patients with hepatocellular carcinoma (HCC) [9, 13]. However, the underlying mechanism of how GTSE1 interacts with other cell cycle-related genes to influence prognosis in liver cancer is still poorly understood. In this study, we integrated RNA microarray analysis with cellular experiments to identify the function of GTSE1 on liver cancer prognosis and found that three other cell cycle-related differentially expressed genes after GTSE1 knockdown could be applied in predicting prognosis of liver cancer.

## 2. Materials and Methods

### 2.1. Bioinformatics Analysis

RNA-Seq data and clinical data were downloaded from the TCGA-LIHC dataset. LinkedOmics [14] was used to analyze the correlation between GTSE1 expression and clinical factors and the correlation between the expression level of MCM6, PCNA, CDC20, and GTSE1. Chi-square tests were conducted between GTSE1 expression and clinical factors. The HCCDB database [15] (http://lifeome.net/database/hccdb) was used to analyze the function of GTSE1, MCM, PCNA, and CDC20 genes in the prognosis of liver cancer patients with a Kaplan-Meier plotter. The Kyoto Encyclopedia of Genes and Genomes (KEGG) and Gene Ontology (GO) enrichment analysis pathway was performed using DAVID (Database for Annotation, Visualization, and Integration Discovery; https://david.abcc.ncifcrf.gov/). STRING (version 11.0) was used to perform the protein interaction network analysis of GTSE1, MCM6, PCNA, and CDC20, which provides scores of combined evaluations of coexpression, experimental/biochemical data, and association in curated databases, and comentioned in PubMed abstracts.

### 2.2. Construction of Effective Lentiviral-Mediated RNAi against GTSE1

Small interfering RNA (siRNA) target sites (psc34526: CTACTCCTACAAATCAATT; psc34528: GCGAGATTCCTGTCTAAAT) were designed against GTSE1 gene (Homo sapiens G2 and S phase expressed 1, mRNA; NCBI Reference Sequence: NM_016426). Double-strand DNA oligonucleotides were then synthesized based on the short hairpin RNA (shRNA) interference sequences and subcloned into lentiviral plasmid GV115 cloning vectors in TOP10 cells (Tiangen, China). Lentiviruses were harvested from 293 T cells into which cotransfected with the recombinant plasmids, pHelper1.0 and pHelper2.0. The virus titer was determined by a fluorescence method. In addition, lentivirus vectors inserted with scramble sequence (TTCTCCGAACGTGTCACGT) were simultaneously prepared as a negative control (shCtrl).

### 2.3. Cell and Cell Culture

Human hepatoma cell BEL-7404 was used in the study. Cells were cultured in Roswell Park Memorial Institute 1640 medium and supplemented with fetal calf serum (FBS, Ausbian, Australia) and 5 *μ*g/mL puromycin (Clontech, USA). BEL-7404 cells were maintained at 37°C with 5% CO_2_.

### 2.4. Transfection and Real-Time qPCR

shRNA lentivirus against GTSE1 was transfected into the BEL-7404 cells using polybrene and enhanced infection solution (Eni.S, GeneChem, China) following the standard procedures. After 72 h, cells transfected with lentiviral-mediated shRNA were harvested, and the GTSE1 knockdown efficiency was examined on the mRNA level using the real-time quantitative polymerase chain reaction (qRT-PCR). Total RNA was extracted with a TRIzol reagent (Pufei, China), converted to cDNA with M-MLV reverse transcriptase enzyme (Promega, USA), and measured with TaKaRa SYBR Master Mixture Kit (TaKaRa, Japan) on an MX3000p Real-time PCR system (Agilent, USA). To normalize the expression of GTSE1, GAPDH was used as the internal control. A 2^−*ΔΔ*CT^ method was used to calculate the relative mRNA level. Triple experiments were performed.

### 2.5. Western Blotting

Total protein was extracted with 2x Lysis Buffer, separated by SDS-PAGE, and transferred to a PVDF membrane with an electroblotting device (Bio-Rad, Richmond, CA). The membrane was blocked with TBST solution containing 5% nonfat milk at room temperature for 1 h. The membrane was then incubated with Mouse Anti-Flag (1 : 2000, Sigma-Aldrich, USA) and Mouse anti-GAPDH (1 : 5000, Santa-Cruz, USA). Then the membrane was washed three times with TBST and incubated with Goat Anti-Mouse IgG (1 : 2000, Santa-Cruz, USA) at room temperature for 2 h. Protein bands were visualized by X-ray imaging with Pierce™ ECL Western Blotting Substrate kit (Thermo Fisher Scientific, USA).

### 2.6. Plate Analysis with the Adherent Cell Cytometry Celigo®

Cells transfected with shRNA lentivirus stably expressed green fluorescence protein (GFP), and the adherent cell cytometry system Celigo® allowed rapid imaging and quantification of cellular fluorescence expression [16]. Cells at the exponential stage were digested with trypsin (Sangon, China), resuspended, and seeded in a 96-well plate. One hundred microliters BEL-7404 was added to each well and incubated at 37°C in 5% CO_2_. After plating, plates were analyzed using Celigo® equipped with green fluorescence channel every 24 h for 5 days. The cell proliferation curves were plotted based on total cell count and proliferation ratio (cell count at each day/cell count at day 1).

### 2.7. Microarray Analysis and Functional Enrichment Analysis

Total RNA extraction was performed with a TRIzol reagent (Pufei, China) according to standard procedures. Samples selected for the microarray analysis must meet the following requirements: 1.7 < A260/A280 < 2.2 (Thermo NanoDrop 2000), RIN ≥ 7.0, and 28S/18S > 0.7 (Agilent 2100 Bioanalyzer). aRNA (amplified RNA) was then obtained via GeneChip 3′IVT PLUS Kit (Affymetrix). Hybridization of the aRNA and the GeneChip® Primeview™ Human Gene Expression Array was conducted with GeneChip Hybridization Wash and Stain Kit. Microarrays were read with GeneChip Scanner 3000. Fold change > 1.5 and *p* < 0.05 were set as the cutoff criterion.

### 2.8. Flow Cytometry for Cell Cycle Analysis

Cells were harvested after trypsinization, and cell suspensions were washed in ice-cold D-Hanks (GeneChem, China). Then the cells were fixed in 75% ice-cold ethanol. For FACS analysis, cell suspension was treated with cell staining solution (40 × 2 mg/mL PI : 100 × 10 mg/mL RNase : 1 × D‐Hanks = 25 : 10 : 1000) for 10 min at 4°C in the dark. Guava easyCyte HT system (Millipore, USA) was used to perform the cell cycle analysis.

### 2.9. Statistical Analysis

All values were presented as the mean ± standard error of the mean. All statistical tests were two-sided. A *p* value of less than 0.05 was considered to be statistically significant.

## 3. Results

### 3.1. GTSE1 High Expression Was Correlated with Poor Prognosis in Liver Cancer

**Figure 1:**
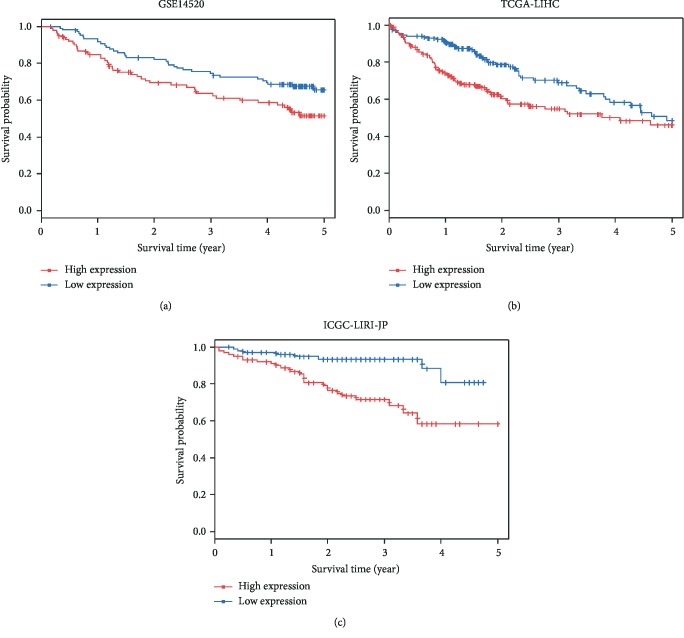
Analysis of GTSE1 gene expression in the prognosis of liver cancer patients using a Kaplan-Meier plotter. Samples were stratified into the high-expression (red) or low-expression (blue) groups by the median expression of GTSE1 gene (cutoff is log2 (1 + TPM) = 6.47) from datasets GSE14520 (a), TCGA-LIHC (b), and ICGC-LIRI-JR (c).

The prognostic value of GTSE1 was identified using bioinformatics analysis. Samples from TCGA-LIHC divided into two groups by the median value of normalized GTSE1 expression signal as the cutoff value were analyzed (Table 1). GTSE1 expression was positively correlated with the stage of primary liver tumor (*p* = 0.0274) and significantly higher in Asian patients (*p* = 0.0098). Upregulated GTSE1 expression in liver cancer was well correlated with a higher pathologic stage with significance (*p* = 0.0098), suggesting that GTSE1 might provide effective prognosis prediction [17]. To further identify the prognostic value of GTSE1 expression in liver cancer, the relationship between GTSE1 expression and overall survival (OS) time in HCC patients was analyzed based on HCCDB, a database of hepatocellular carcinoma expression atlas which contains 15 public HCC expression datasets with around 4000 clinical samples [15]. The prognostic value of GTSE1 was investigated using three datasets: GSE14520, TCGA-LIHC, and ICGC-LIRI-JP (Figure 1). GSE14520 is a cohort of 64 HCC patients in the USA. The Cancer Genome Atlas Liver Hepatocellular Carcinoma (TCGA-LIHC) cohort was composed of 768 HCC patients of all races. The third dataset, Liver Cancer-RIKEN, JP (ICGC-LIRI-JP), is a cohort of 260 HCC patients in Japan. In all datasets, HCC samples were classified into the high-/low-expression groups by the median expression value of GTSE1 (cutoff is log2 (1 + TPM) = 6.47). Kaplan-Meier curves and log-rank test indicated that high or low level of GTSE1 expression represented significant different prognosis in all 3 HCC datasets: GSE14520 (*p* < 0.05, Figure 1(a)), TCGA-LIHC (*p* < 0.01, Figure 1(b)), and ICGC-LIRI-JP (*p* < 0.001, Figure 1(c)). In ICGC-LIRI-JP, the OS rate of patients with a high GTSE1 expression was markedly lower in comparison with those with low GTSE1 expression.

### 3.2. Deficiency of GTSE1 Inhibited Human Hepatoma Cell Proliferation

**Figure 2:**
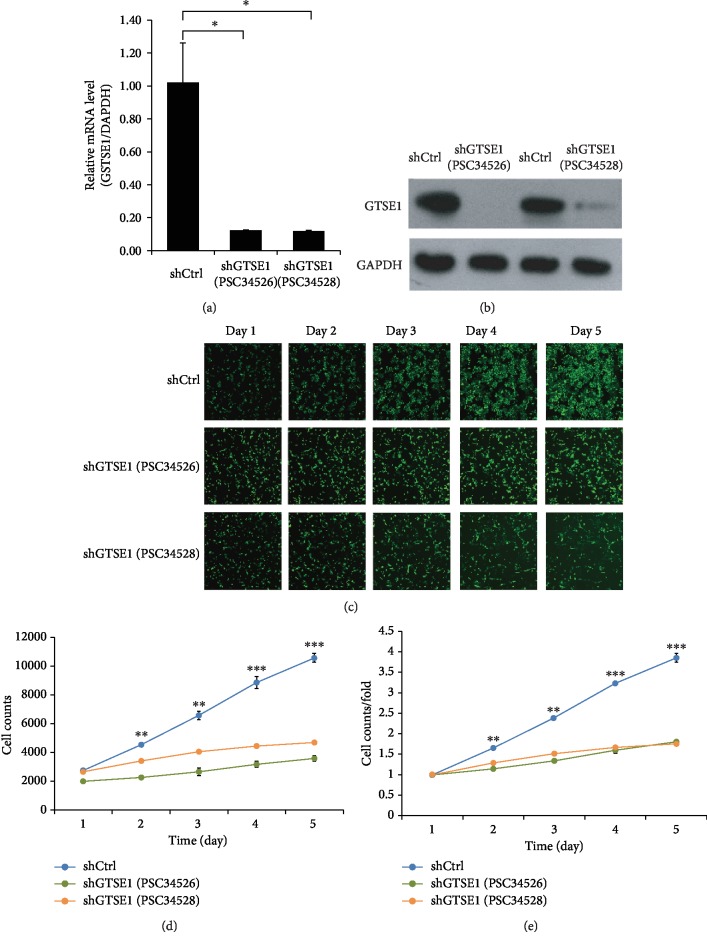
Cell proliferation of Bel-7404 cells with GTSE1 knockdown. (a) Expression level of GTSE1 mRNA using real-time quantitative PCR and Western blotting. Triple experiments were performed. GAPDH was used as the endogenous control. Values were represented as the mean ± SD. ^∗^*p* < 0.05. (b) Western blotting of GTSE1 protein. (c) Representative images of BEL-7404 cells in control and shRNA infection from day 1 to day 5. (d, e) Cell proliferation after GTSE1 knockdown was determined by the Celigo image cytometer for 5 days. (d) Cell count curves of shGTSE1 (psc34526) and shGTSE1 (psc34528) and the control group (shCtrl) over time. (e) Curves of fold change of the cell count of shGTSE1 (psc34526) and shGTSE1 (psc34528) and the control group (shCtrl) over time. Triplicate for each group; values were represented as the mean ± SD. The cell proliferation rate of the control group is significantly higher than those in the shGTSE1 groups (*p* < 0.001). The differences between the control group (shCtrl) and shGTSE1 (psc34526) were analyzed by a *t*-test. ^∗∗^*p* < 0.01 and ^∗∗∗^*p* < 0.0001.

To investigate GTSE1 cellular functions in HCC, GTSE1 was knocked down in HCC cell line Bel-7404 using lentiviral-mediated shRNA. Two kinds of shRNA strands, psc34526 and psc34528, were designed and tested for better GTSE1 silencing performance (Supplementary [Supplementary-material supplementary-material-1]). Over 80% of cells in both the psc34526 and psc34528 groups were GFP positive, indicating good infection efficiencies after 72 h transfection (Supplementary [Supplementary-material supplementary-material-1]). Decreased expression of GTSE1 in Bel-7404 cells at the mRNA level (*p* < 0.05) and protein level (*p* < 0.01) was validated using real-time quantitative PCR and Western blotting, respectively (Figures 2(a) and 2(b)). The knockdown efficiency of psc34526 (87.8%) and that of psc34528 (88.2%) were comparable, so both strands were applied in further assays except additional illustration (Supplementary [Supplementary-material supplementary-material-1]).

Cellular functions of Bel-7404 cells with GTSE1 knockdown (psc34526 and psc34528) were compared with those of cells transfected with control strand at proliferation, metastasis, invasion, and apoptosis level. Cells in culture were imaged and counted based on GFP fluorescence for 5 days (Figure 2(c)). Cell proliferation rate was inhibited at 60 to 70 percent (*p* < 0.01) compared with that in the control group as shown in the proliferation curve (Figures 2(d) and 2(e)), which was also validated using MTT assay (Supplementary [Supplementary-material supplementary-material-1]). In addition, GTSE1 knockdown Bel-7404 cells showed lower metastasis rates (Supplementary [Supplementary-material supplementary-material-1]) and lower invasion rates (Supplementary [Supplementary-material supplementary-material-1]). Their capacity for colony formation decreases (Supplementary [Supplementary-material supplementary-material-1]). Meanwhile, both the apoptosis percentage and caspase3/7 activity in the shGTSE1 group increased (Supplementary [Supplementary-material supplementary-material-1]). In sum, Bel-7404 cells with GTSE1 knockdown exhibited fewer characteristics of cancer cells.

### 3.3. 979 DEGs Were Identified in GTSE1 Knockdown Human Hepatoma Cell

**Figure 3:**
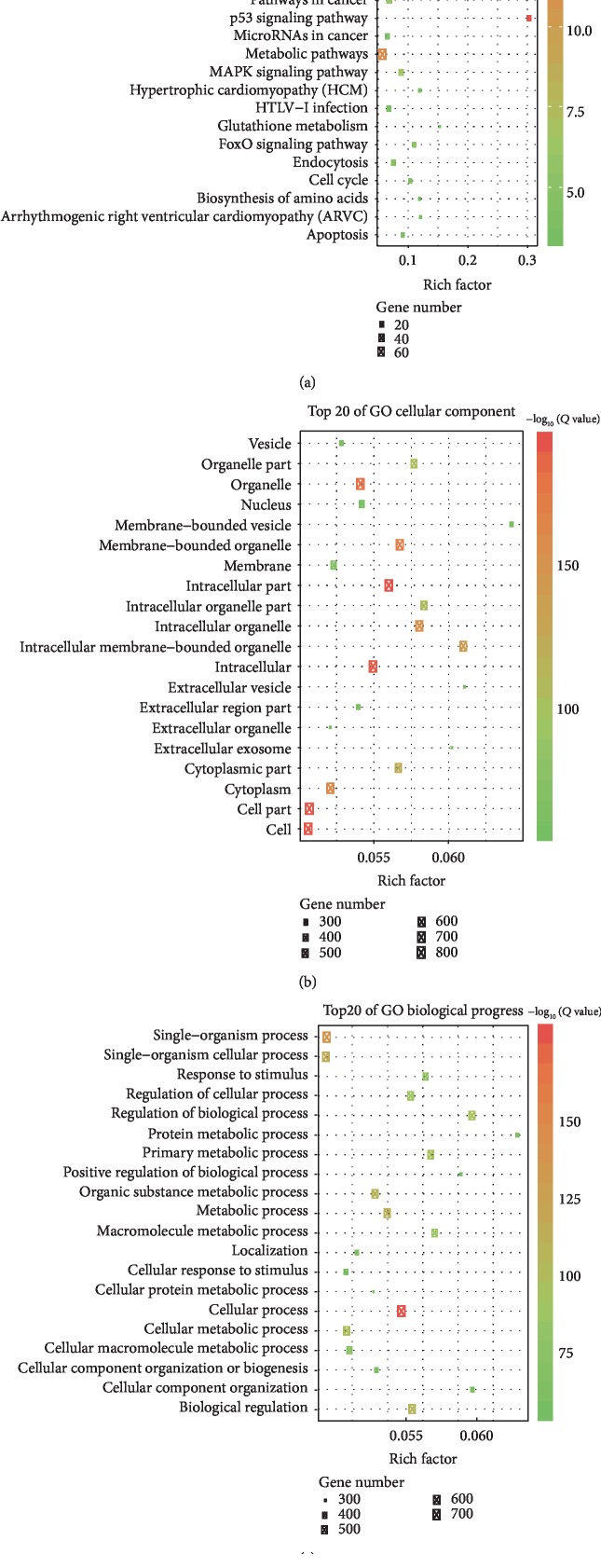
Functional and pathway enrichment analyses in DEGs. (a) Top 20 KEGG-enriched gene pathways of DEGs. (b) Top 20 GO pathways of DEGs on the cellular component. (c) Top 20 GO pathways of DEGs on the biological progress. The size of the rectangles represents gene numbers in each term, and the colors represent minus logarithms of adjusted *p* values.

Transcriptome analysis showed that between BEL-7404 cells with GTSE1 knockdown (shGTSE1, psc34526, and psc34528) and control group (shCtrl), 979 genes were identified to be differentially expressed genes (∣fold change∣ > 1.5, *p* < 0.05) in shGTSE1-BEL-7404, including 459 activated genes and 520 repressed genes. Functional characteristics of the identified DEGs using KEGG pathway analysis showed that the DEGs were enriched in the p53 signaling pathway, glutathione metabolism, and protein processing in endoplasmic reticulum (Figure 3(a)). GO analysis under cellular component (CC) and biological progress (BP) of DEGs are shown in Figures 3(b) and 3(c). In CC, the identified DEGs could act through membrane-bounded organelle, intracellular membrane-bounded organelle, and extracellular vesicle. The most observably enriched BP of DEGs were protein metabolic process, regulation of biological process, and cellular component organization.

### 3.4. Cell Cycle Dysregulation in GTSE1 Knockdown Human Hepatoma Cell Was Validated

**Figure 4:**
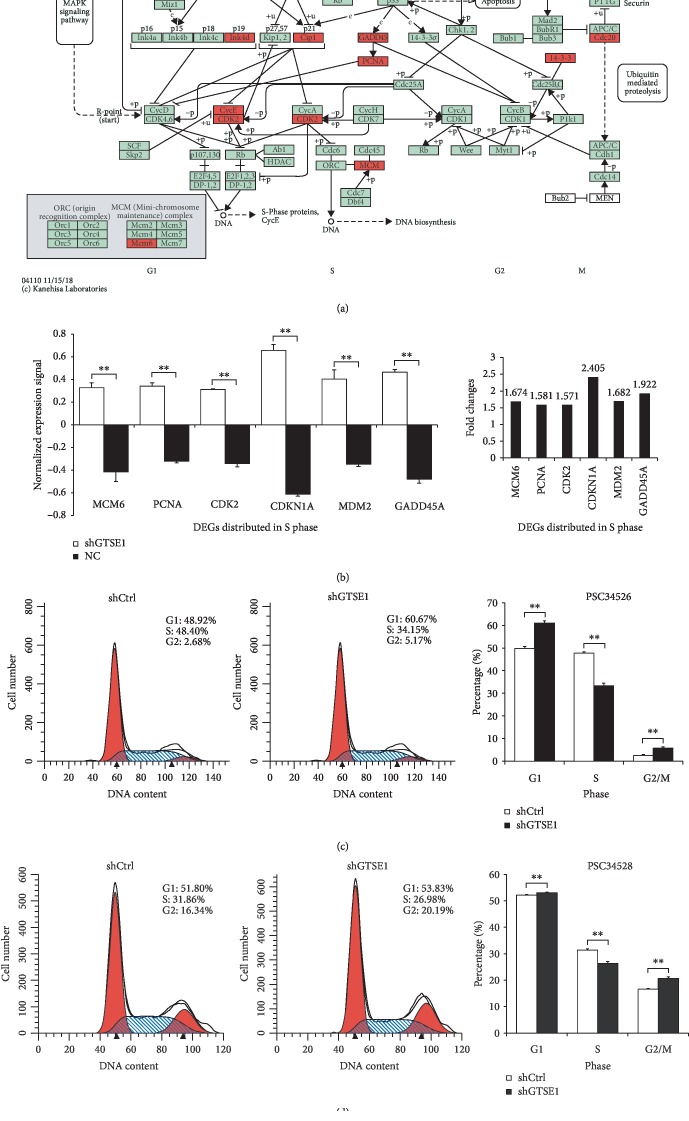
An interaction network of the DEGs in cell cycle and detection of the effect of GTSE1 knockdown on the cell cycle distribution. (a) An interaction network of DEGs related to cell cycle regulation. Red represented DEGs detected in microarray analysis including TGF*β*, Ink4d, CycE, CDK2, Cip1, Mdm2, GADD45, PCNA, MCM (Mcm6), ATMATF, 14-3-3, and Cdc20. (b) Normalized expression level and fold change of 6 DEGs distributed in S phase in shGTSE1 cells. NC: negative control. (c, d) FACS-based DNA content analysis was used to explore the effect of GTSE1 knockdown on the cell cycle profiles of Bel-7404 cells. The percentage of cells in G1, S, and G2/M phases is shown. Data are presented as the means ± SD. ^∗∗^*p* < 0.01 and ^∗∗∗^*p* < 0.0001.

A functional enrichment analysis-based interaction network (Figure 4(a)) showed that 14 DEGs marked red are involved in cell cycle-related pathways. Six of them were mainly distributed in S phase of cell division (MCM6, PCNA, Mdm2, GADD45A, Cip1, and CDK2) and were all upregulated in shGTSE1 cells (Figure 4(b)). The effects of these DEGs in cell cycle were assessed by FACS on shCtrl and shGTSE1 cells. Full data of cell cycle analysis are provided in Supplementary [Supplementary-material supplementary-material-1]. As shown in Figures 4(b) and 4(c), shGTSE1 cells (psc34526 and psc34528) in S phase decreased significantly (*p* < 0.001, *p* < 0.01) with stalling of cells in G2/M phase and G1 phase (*p* < 0.01) compared with the shCtrl group, which were consistent with expectation based on the interaction network. Therefore, GTSE1 knockdown affected the G1-to-S or S-to-G2 transition of the cell cycle.

### 3.5. Additional GTSE1-Interacted DEGs Were Related to Liver Cancer Prognosis

**Figure 5:**
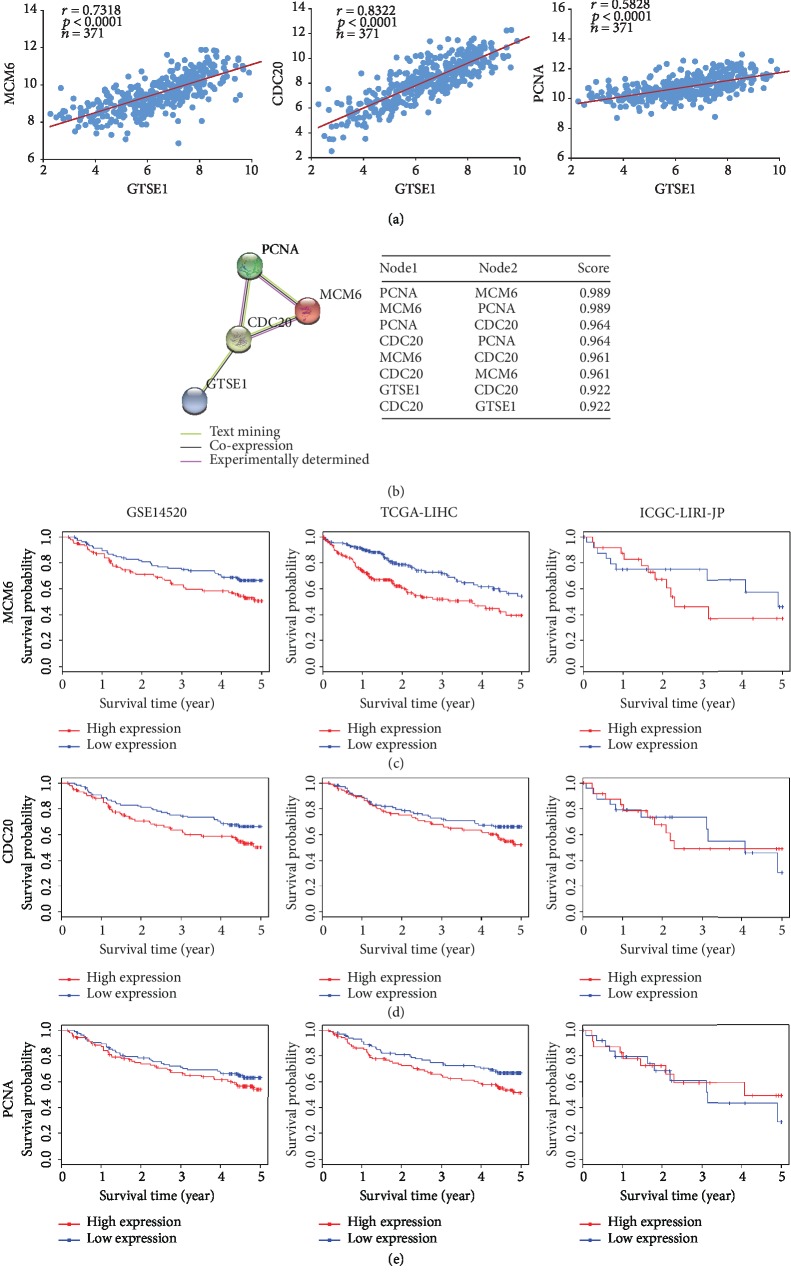
Association between 3 DEGs regulating cell cycle and survival of liver cancer patients. (a) Pearson's pairwise correlation plots of the expression level of MCM6, CDC20, and PCNA vs GTSE1. (b) Protein interaction network and interaction scores among GTSE1, MCM6, CDC20, and PCNA. (c–d) The Kaplan-Meier curves of overall survival (OS) stratified by altered expression of MCM6 (a), CDC20 (b), and PCNA (c) gene from datasets GSE14520, TCGA-LIHC, and ICGC-LIRI-JR.

Coexpression levels of three cell cycle-related DEGs (MCM6, CDC20, and PCNA) and GTSE1 were evaluated. Based on TCGA-LIHC dataset and LinkedOmics, Pearson's pairwise correlations were plotted for all liver cancer patients (Figure 5(a)). The mRNA expression levels of MCM6, CDC20, and PCNA are all positively correlated to GTSE1 (Pearson's correlation coefficient = 0.7318, 0.8722, and 0.5828, respectively), suggesting that the three DEGs may have a synergistic effect with GTSE1. Among the three DEGs, the expression level of CDC20 is the most significant one correlated with GTSE1.

To investigate possible correlations between four genes, the protein interaction network (Figure 5(b)) of GTSE1, MCM6, CDC20, and PCNA was built with STRING. The PPI enrichment *p* value of the network is 0.00304, which means the four proteins are at least partially biologically connected as one group. Among DEG-transcribed proteins, the score between CDC20 and GTSE1 was the highest, suggesting the most significant functional link. The interactions between CDC20, MCM6, and PCNA have been experimentally determined. Therefore, GTSE1 might influence the cell cycle progression synergistically with CDC20, MCM6, and PCNA.

MCM6, CDC20, and PCNA are also associated with prognosis in liver cancer. Samples were divided into the high-expression group and low-expression group by median expression levels of MCM6, CDC20, and PCNA in three datasets from the HCCDB database (cutoffs are 9.53, 8.15, and 10.79, respectively). Patients with high MCM6 (Figure 5(c)), CDC20 (Figure 5(d)), or PCNA (Figure 5(e)) protein expression presented unfavorable prognosis which was consistent with that of GTSE1 in Figure 1.

## 4. Discussion

GTSE1 is a microtubule-localized cell cycle-related protein. It regulates microtubule stability by inhibiting MCAK (mitotic centromere-associated kinesin), which has potent depolymerase activity. It also modulates p21^CIP1/WAF1^ stability whose expression is critical for cell cycle control [18]. Although previous researches have demonstrated the prognostic value of GTSE1 in various cancers, its underlying mechanisms in liver cancer remain to be characterized. In this study, we generated GTSE1 knockdown HCC cells and conducted microarray analysis. 979 DEGs were identified, and they were engaged in the p53 signaling pathway and protein processing in endoplasmic reticulum, membrane-bounded organelle, and cellular component organization.

Three cell cycle-related DEGs (*CDC20*, *PCNA*, and *MCM6*) were significantly coexpressed with *GTSE1*, and they may synergistically affect regulations in the cell cycle. The cell division cycle 20 homolog (CDC20) is an essential cofactor controlling chromosome segregation and mitotic exit [19]. Similar to GTSE1, it regulates the stability of phosphorylated MCAK in metaphase-anaphase transition [20]. Proliferating cell nuclear antigen (PCNA) is a 36 kDa protein, which acts in conjunction with human DNA polymerase *δ* in both DNA duplication and DNA-repair [21–23]. It cooperates with p21 in the cell cycle [22]. Therefore, GTSE-1 might mediate the stabilization of p21 to protect microtubule, and then DNA-bound p21 together with PCNA participates in DNA repair. The MCM family shares a role in eukaryotic genome replication through functioning as a helicase in replication elongation [24]. GTSE1 binds to the microtubule plus-end tracking protein EB1 in cancer [11] and regulates microtubule stability for accurate chromosome alignment and segregation [4]. Microtubules bind directly to minichromosome instability 12 (MIS12) which is the core protein required for maintaining structural integrity of mitotic kinetochore [25]. Thus, GTSE1 may develop combined action with MCM in mediating sister kinetochore cohesion for reduction division. It has previously been shown that preventing cyclin-dependent kinases from recruiting MCM to chromatin would result in centrosome overduplication without passage through mitosis [26]. Centrosomes are microtubule-assembly centers, and GTSE1 might modulate centrosomal microtubule formation in an MCAK-dependent manner.

GTSE1, together with CDC20, PCNA, and MCM, presented unfavorable prognosis of liver cancer in our study. Emerging evidence indicated abnormal expression of genes in cell cycle, and apoptosis regulation, like p53, Rb, p27, and TGF*β*/IGF2R, is related to etiopathogenesis and prognosis of liver cancer patients [1]. GTSE1 has been demonstrated as a potential biomarker for poor clinical outcome in several types of cancer [10, 13, 27]. Previous research suggested GTSE1's functional role in hepatocellular carcinoma (HCC) proliferation, colony formation, invasion, and overall survival [9, 13]. In our study, DEGs in GTSE1 knockdown HCC cells were enriched in critical steps of oncogenesis including dysregulated cell cycle and unlimited cell growth, which might account for the adverse cancer prognosis.

In addition to GTSE1, overexpression of CDC20, PCNA, and MCM has been detected in many types of human cancer [28–30]. In the MCM family, abnormal expression of MCM2, MCM3, MCM4, MCM5, and MCM7 also results in reducing prognosis in liver cancer patients according to the HCCDB database [15]. The minichromosome maintenance protein (MCM) family is implicated in the control of eukaryotic genome replication and proves to be useful markers for tumor proliferation [24]. Several studies demonstrated overexpression of MCM6 also predicts poor survival in patients with several types of cancer, including HCC [31–34]. Future studies should attempt to clarify the underlying mechanism of GTSE1 together with minichromosome-related protein-mediated DNA replication and cancer prognosis.

## 5. Conclusions

The aim of the present study was to elucidate the role of GTSE1 in the prognosis of liver cancer and to find potential prognostic indicators in liver cancer. In this study, we found that increased GTSE1 expression contributed to advanced pathologic stage and poor prognosis in liver cancer patients. Consistent with previous research, downregulated GTSE1 was verified to lead to decreased cell proliferation and cell cycle arrest. Furthermore, 979 genes correlated with cellular component organization and biological process regulation are differentially expressed with GTSE1 knockdown. Among the DEGs, CDC20, PCNA, and MCM6 might synergistically affect regulations in cell cycle with GTSE1 and might be potential prognostic predictors in liver cancer. Our study provides a valuable resource for molecular mechanism of liver cancer progression and prognosis prediction. Future studies validating the molecular mechanism underlying tumorigenesis and prognosis of liver cancer could emphasize the association between GTSE1 and minichromosome maintenance protein (MCM) family.

## Figures and Tables

**Table 1:** GTSE1 expression and clinical factors.

Clinical factor	Cases	GTSE1 median	GTSE1 expression		*P* value
			Low	High	
Age (years)					
<40	29	6.251	14	15	0.846
≥40	311		156	155	
Ethnicity					
Hispanic or Latino	17	6.492	7	10	0.461
Not Hispanic or Latino	310		156	154	
Race					
White	169	6.463	97	72	**0.010**
Black or African American	15		9	6	
Asian	148		59	89	
American Indian or Alaska Native	1		1	0	
Tumor purity					
<0.6	28	6.455	11	17	0.242
≥0.6	315		160	155	
Residual tumor					
r0	303	6.397	151	152	0.586
r1	15		7	8	
r2	1		1	0	
Pathologic stage					
Stage i	161	6.463	92	69	**0.010**
Stage ii	77		34	43	
Stage iii	80		31	49	
Stage iv	3		3	0	
Pathology T stage					
t1	168	6.465	97	71	**0.027**
t2	84		37	47	
t3	75		29	46	
t4	13		7	6	
Pathology N stage					
n0	239	6.593	120	119	0.561
n1	3		1	2	
Pathology M stage					
m0	245	6.514	121	124	0.081
m1	3		3	0	

Cases from TCGA-LIHC were divided into the GTSE1 low and high groups by the median value of normalized GTSE1 expression signal as the cutoff value. *P* values with significance were shown in bold.

## Data Availability

The datasets analyzed for this study can be found in the HCCDB database (http://lifeome.net/database/hccdb). Expression data of GTSE1 knockdown hepatoma cell line Bel-7404 is available at https://www.ncbi.nlm.nih.gov/geo/query/acc.cgi?acc=GSE130083.

## Supplementary Materials

Supplementary Materials Supplementary Figure 1: images of BEL-7404 cells transfected by shRNA lentivirus against GTSE1. (A) Transfection image of psc34526. (B) Transfection image of psc34528. Over 80% cells in both the shGTSE1 (psc34526) and shGTSE1 (psc34528) groups were infected after 72 hours after transfection. Supplementary Figure 2: cell growth rates measured by MTT assay. (A) Absorption value and relative fold changes of shCtrl and shGTSE1 (psc34526) at 490 nm. (B) Absorption value and relative fold changes of shCtrl and shGTSE1 (psc34528) at 490 nm. For A and B, OD 490 represents the number of living cells. Data are presented as the mean ± SD. GAPDH was used as the endogenous control. Supplementary Figure 3: colony formation of BEL-7404 cells with GTSE1 knockdown. (A) Image and colony number evaluation of shGTSE1 (psc34526). (B) Image and colony number evaluation of shGTSE1 (psc34528). For A and B, data are presented as the mean ± SD. ^∗∗^*p* < 0.01 vs. shCtrl. GAPDH was used as the endogenous control. Supplementary Figure 4: results of apoptosis assay and caspase3/7 assay. (A) Apoptosis percentage of shGTSE1 (psc34526), shGTSE1 (psc34526), and shCtrl 5 days after RNAi transfection. (B) Caspase3/7 activity between shGTSE1 (psc34526), shGTSE1 (psc34526), and shCtrl 5 days after RNAi transfection. For A and B, data are presented as the mean ± SD. ^∗^*p* < 0.05 and ^∗∗^*p* < 0.01 vs. shCtrl. GAPDH was used as the endogenous control. Supplementary Table 1: sequences of shRNA against GTSE1 Supplementary Table 2: efficiency of shRNA knockdown against GTSE1. Supplementary Table 3: cell counts of transwell assay (200x). Supplementary Table 4: cell counts of invasion assay (200x). Supplementary Table 5: data of cell cycle analysis.
