# Supplementary material for: GTSE1, CDC20, PCNA, and MCM6 Synergistically Affect Regulations in Cell Cycle and Indicate Poor Prognosis in Liver Cancer
Source: Anal Cell Pathol (Amst). 2019 Dec 30;2019:1038069. doi: 10.1155/2019/1038069 (PMC7012210; doi:10.1155/2019/1038069)
Supplement: Supplementary Materials — Supplementary Figure 1: images of BEL-7404 cells transfected by shRNA lentivirus against GTSE1. (A) Transfection image of psc34526. (B) Transfection image of psc34528. Over 80% cells in both the shGTSE1 (psc34526) and shGTSE1 (psc34528) groups were infected after 72 hours after transfection. Supplementary Figure 2: cell growth rates measured by MTT assay. (A) Absorption value and relative fold changes of shCtrl and shGTSE1 (psc34526) at 490 nm. (B) Absorption value and relative fold changes of shCtrl and shGTSE1 (psc34528) at 490 nm. For A and B, OD 490 represents the number of living cells. Data are presented as the mean ± SD. GAPDH was used as the endogenous control. Supplementary Figure 3: colony formation of BEL-7404 cells with GTSE1 knockdown. (A) Image and colony number evaluation of shGTSE1 (psc34526). (B) Image and colony number evaluation of shGTSE1 (psc34528). For A and B, data are presented as the mean ± SD. ∗∗p < 0.01 vs. shCtrl. GAPDH was used as the endogenous control. Supplementary Figure 4: results of apoptosis assay and caspase3/7 assay. (A) Apoptosis percentage of shGTSE1 (psc34526), shGTSE1 (psc34526), and shCtrl 5 days after RNAi transfection. (B) Caspase3/7 activity between shGTSE1 (psc34526), shGTSE1 (psc34526), and shCtrl 5 days after RNAi transfection. For A and B, data are presented as the mean ± SD. ∗p < 0.05 and ∗∗p < 0.01 vs. shCtrl. GAPDH was used as the endogenous control. Supplementary Table 1: sequences of shRNA against GTSE1 Supplementary Table 2: efficiency of shRNA knockdown against GTSE1. Supplementary Table 3: cell counts of transwell assay (200x). Supplementary Table 4: cell counts of invasion assay (200x). Supplementary Table 5: data of cell cycle analysis. [file 1038069.f1.pdf]

GTSE1, CDC20, PCNA and MCM6 Synergistically Effect in Cell Cycle and Indicate Poor Prognosis in Liver Cancer

Supplementary Materials

YONGCHANG ZHENG [1, *], YUE SHI [1, *], SI YU [1, *], YUANYUAN HAN[2], KAI KANG [1], SHUNDA DU[1], HUAJIAN GU[3], XINTING SANG [1], YANG CHEN [4, #] AND JINGYU WANG[4, 5 #]

[1]Department of Liver Surgery, Peking Union Medical College Hospital, Chinese Academy of Medical Sciences & Peking Union Medical College, Beijing 100730, China;

[2]Center of Tree Shrew Germplasm Resources, Institute of Medical Biology, The Chinese Academy of Medical Sciences and Peking Union Medical College, Yunnan Key Laboratory of Vaccine Research and Development on Severe Infectious Diseases, Kunming, Yunnan 650118, P.R. China.

[3]Department of Hepatobiliary Surgery, Affiliated Hospital of Guizhou Medical University, No. 28, Guiyi Street, Yunyan District, 550004 Guiyang P. R. China.

[4]MOE Key Laboratory of Bioinformatics, Center for Synthetic and Systems Biology, Bioinformatics Division, BNRist, Department of Automation, Tsinghua University, Beijing 100084, China.

[5]Department of Basic Medical Science, Tsinghua-Peking Joint Center for Life Sciences, School of Medicine, Tsinghua University, Beijing 100084, China.

*These authors contributed equally to this work.

[#]Correspondence:
Correspondence and requests for materials should be addressed to Y.C. (email: yc@tsinghua.edu.cn) or to J.Y.W. (email: wangjingyu@tsinghua.edu.cn)

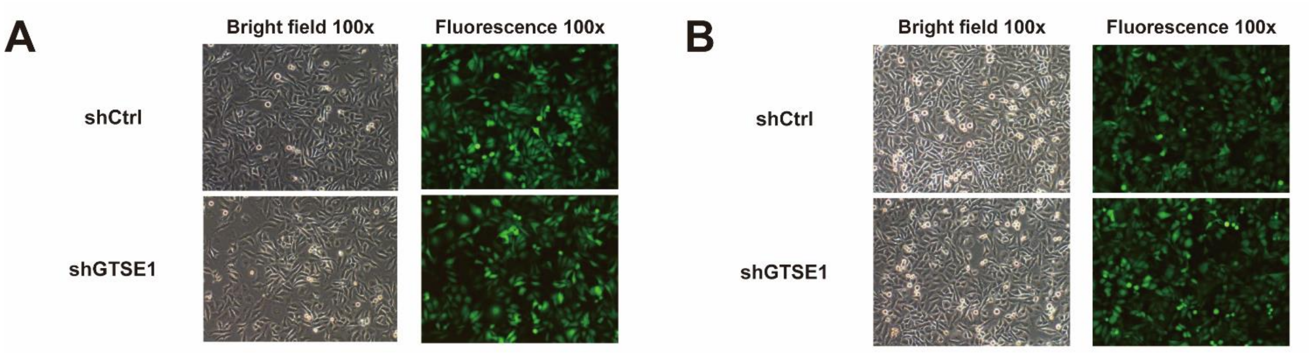

Supplementary Figure 1. Transfection images of BEL-7404 cells transfected by shRNA lentivirus against GTSE1. **(A)** Transfection image of psc34526. **(B)** Transfection image of psc34528. Over 80% cells in both shGTSE1 (psc34526) and shGTSE1 (psc34528) group were infected after 72 hours after transfection.

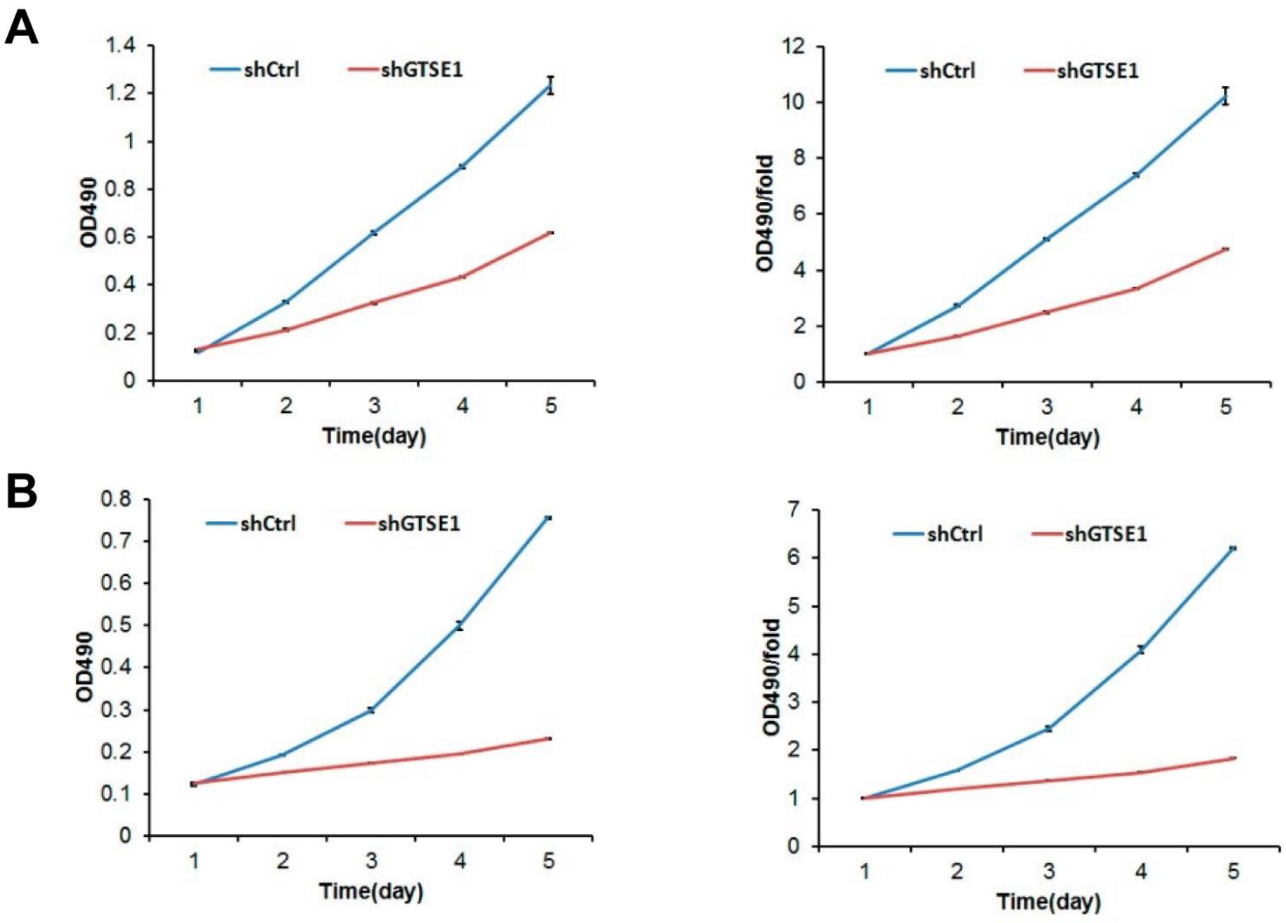

Supplementary Figure 2. Cell growth rates measured by methyl-thiazolyl-tetrazolium (MTT) assay. **(A)** Absorption and absorption fold changes of shCtrl and shGTSE1 (psc34526) at 490 nm. **(B)** Absorption and absorption fold changes of shCtrl and shGTSE1 (psc34528) at 490 nm. For **A** and **B**, OD 490 represents the number of living cells, data are presented as the mean ±SD. GAPDH was used as the endogenous control.

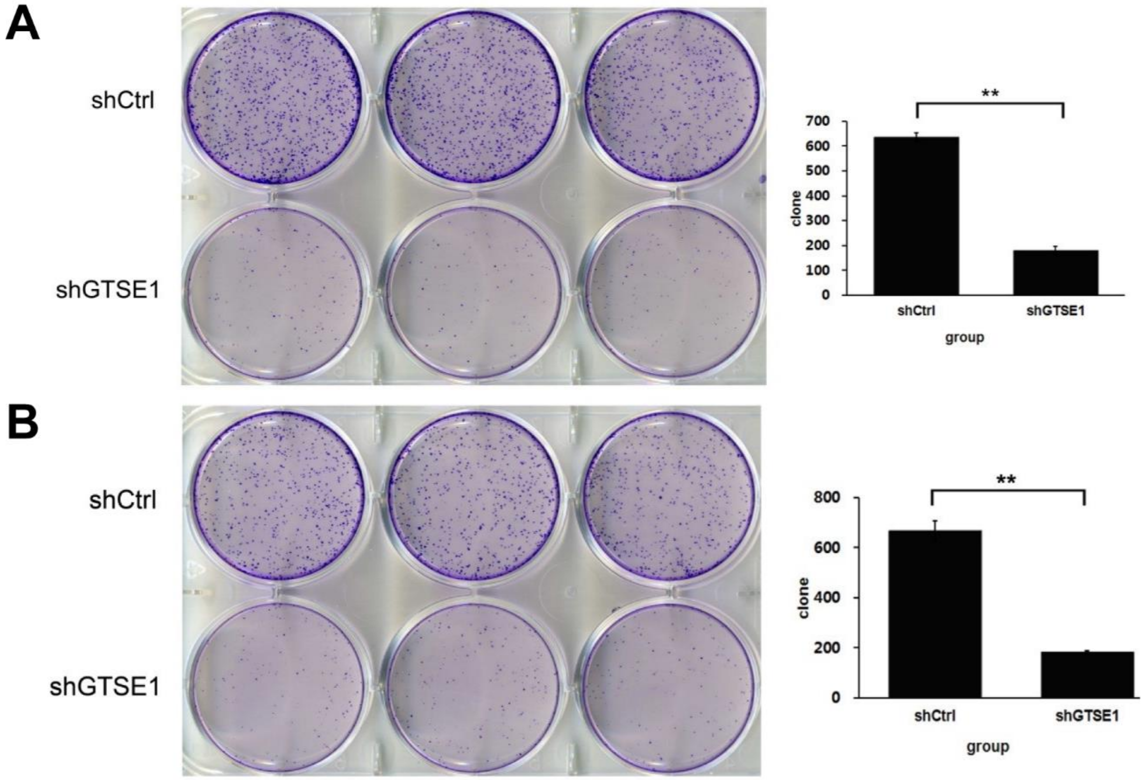

Supplementary Figure 3. Colony formation of BEL-7404 cells with GTSE1 knockdown. **(A)** Image and colony number evaluation of shGTSE1 (psc34526). **(B)** Image and colony number evaluation of shGTSE1 (psc34528). For **A** and **B**, data are presented as the mean ±SD. $^{**}p < 0.01$ vs. shCtrl. GAPDH was used as the endogenous control.

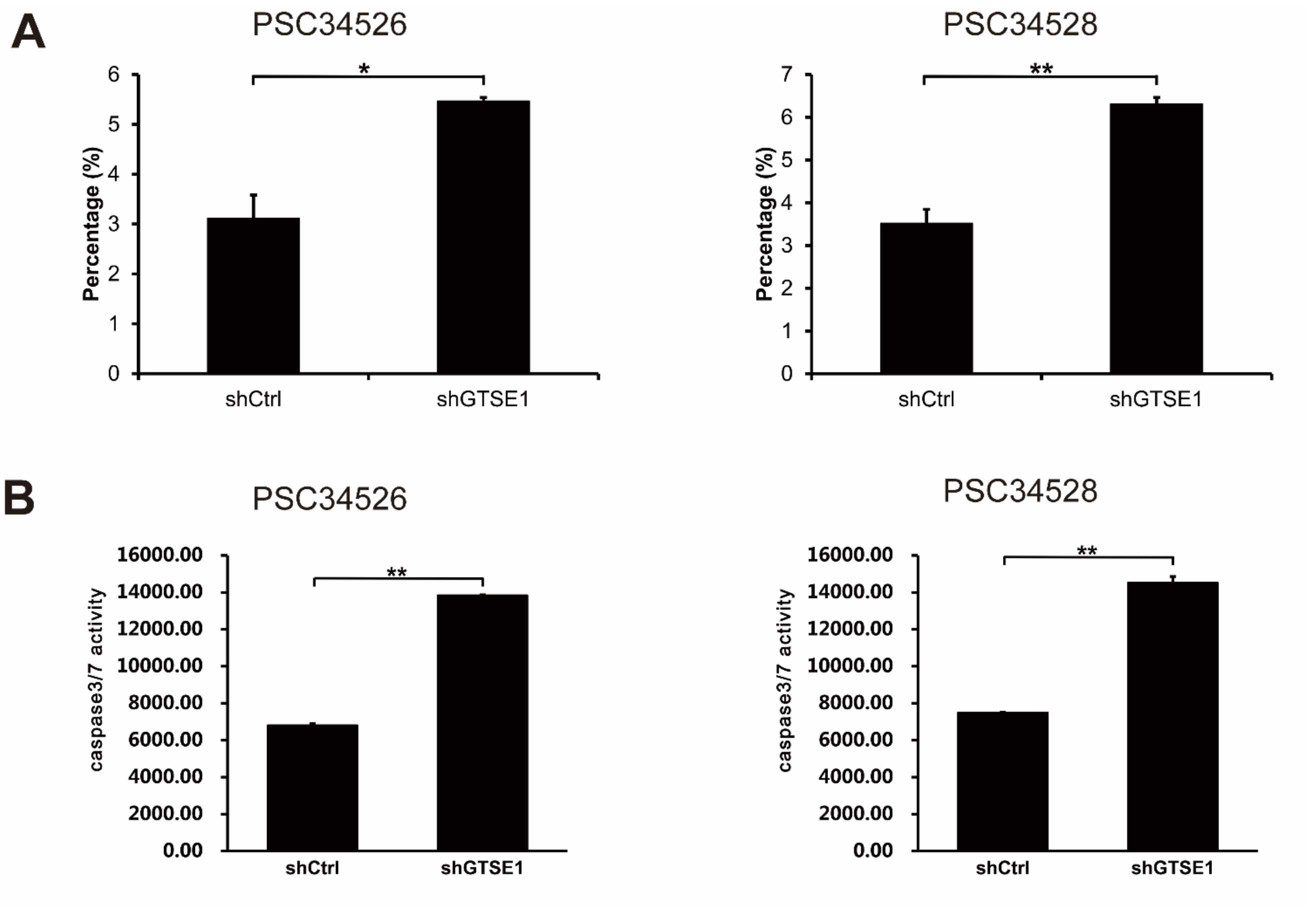

Supplementary Figure 4. Results of apoptosis assay and caspase3-7 assay. **(A)** Apoptosis percentage of shGTSE1 (psc34526), shGTSE1 (psc34526) and shCtrl 5 days after RNAi transfection. **(B)** Caspase3/7 activity between shGTSE1 (psc34526), shGTSE1 (psc34526) and shCtrl 5 days after RNAi transfection. For **A** and **B**, data are presented as the mean ±SD. **p* < 0.05 and ***p* < 0.01 vs. shCtrl. GAPDH was used as the endogenous control.

Supplementary Table 1. Sequences of shRNA against GTSE1

Sequence number	5’ additional bases	STEM	Loop	STEM	3’ additional bases
psc34526-1	CCGG	GCCTACTCCTA<br>CAAATCAATT	CTCGAG	AATTGATTTGT<br>AGGAGTAGGC	TTTTTG
psc34526-2	AATTCAAA<br>AA	GCCTACTCCTA<br>CAAATCAATT	CTCGAG	AATTGATTTGT<br>AGGAGTAGGC	
psc34528-1	CCGG	CGGCGAGATT<br>CCTGTCTAAAT	CTCGAG	ATTTAGACAGG<br>AATCTCGCCG	TTTTTG
psc34528-2	AATTCAAA<br>AA	CGGCGAGATT<br>CCTGTCTAAAT	CTCGAG	ATTTAGACAGG<br>AATCTCGCCG	

This is a provisional file, not the final typeset article

Supplementary Table 2. Efficiency of shRNA knockdown against GTSE1

Sample	Average ($2^{-\Delta\Delta Ct}$)	STDEV	P-value
shCtrl	1.018	0.241	
shGTSE1(PSC34526)	0.122	0.001	0.023
shGTSE1(PSC34528)	0.118	0.005	0.023

Supplementary Table 3. Cell counts of transwell assay (200×)

200X Cell counts	shCtrl			shGTSE1		
	Hole 1	Hole 2	Hole 3	Hole 1	Hole 2	Hole 3
**field 1**	198	217	207	102	112	108
**field 2**	186	210	194	98	108	107
**field 3**	211	197	208	115	119	112
**field 4**	217	219	202	99	111	99
**field 5**	227	199	215	90	109	118
**field 6**	201	187	210	97	108	102
**field 7**	209	196	226	107	98	120
**field 8**	229	227	219	108	118	119
**field 9**	216	192	206	106	101	91
**Migratory cells per field**	210	205	210	102	109	108
**Average**	208			107		
**Stdev.**	3.01			3.75		
**P VALUE**	3.32026E-06					
**Migration Fold Change**	1.01	0.98	1.01	0.49	0.52	0.52
**Average**	1.00			0.51		
**Stdev.**	0.01			0.02		
**P VALUE**	3.32026E-06					

Supplementary Table 4. Cell counts of invasion assay (200×)

	shCtrl	shGTSE1

200X Cell counts	Hole 1	Hole 2	Hole 3	Hole 1	Hole 2	Hole 3
**field 1**	164	164	147	117	101	101
**field 2**	172	177	151	124	98	87
**field 3**	182	172	158	117	98	97
**field 4**	166	181	150	120	87	103
**field 5**	168	188	155	112	107	111
**field 6**	152	172	147	101	104	102
**field 7**	170	171	141	121	97	121
**field 8**	155	173	160	107	103	107
**field 9**	161	169	139	107	105	96
**Migratory cells per field**	**166**	**174**	**150**	**114**	**100**	**103**
**Average**	163			106		
**Stdev.**	12.34			3.75		
**P VALUE**	0.002284129					
**Migration Fold Change**	1.01	1.07	0.92	0.70	**0.61**	**0.63**
**Average**	1.00			0.65		
**Stdev.**	0.08			0.05		
**P VALUE**	0.002284129					

Supplementary Table 5. Data of Cell Cycle analysis

**PSC34526**				**PSC34528**			
**Group**	**G1**	**S**	**G2/M**	**Group**	**G1**	**S**	**G2/M**
**shCtrl**	48.92	48.4	2.68	**shCtrl**	51.8	31.86	16.34
	50.6	47.41	2		52.39	30.69	16.92
	49.79	47.31	2.9		52.1	31.4	16.5
**shGTSE1**	60.67	34.15	5.17	**shGTSE1**	52.83	26.98	20.19
	60.27	33.64	6.09		53.13	25.58	21.29
	62.12	31.96	5.92		53.1	26.42	20.48
**Average**	**G1**	**S**	**G2/M**	**Average**	**G1**	**S**	**G2/M**
**shCtrl**	49.77	47.71	2.53	**shCtrl**	52.1	31.32	16.59
**shGTSE1**	61.02	33.25	5.73	**shGTSE1**	53.02	26.33	20.65
**STDEV**	**ST.G1**	**ST.S**	**ST.G2/M**	**STDEV**	**ST.G1**	**ST.S**	**ST.G2/M**

This is a provisional file, not the final typeset article

**shCtrl**	0.8402	0.6025	0.4692	**shCtrl**	0.295	0.5894	0.2996
**shGTSE1**	0.9734	1.1459	0.4895	**shGTSE1**	0.1652	0.7047	0.5701
**T-Test**	**P Value**			**T-Test**	**P Value**		
	**G1**	**S**	**G2/M**		**G1**	**S**	**G2/M**
**shCtrl vs shGTSE1**	0.00011	0.00004	0.00122	**shCtrl vs shGTSE1**	0.00911	0.00071	0.0004
